# Pyogranulomatous lymphadenitis with Splendore–Hoeppli phenomenon caused by *Neisseria* species in a domestic shorthair cat

**DOI:** 10.1093/jvimsj/aalag076

**Published:** 2026-06-03

**Authors:** Myles McKenna, Finola C Leonard, Hanne Jahns, Kevin Murtagh

**Affiliations:** Global Medical Affairs, Zoetis Inc. , Loughlinstown, Dublin, Ireland; School of Veterinary Medicine, University College Dublin, Dublin, Ireland; School of Veterinary Medicine, University College Dublin, Dublin, Ireland; School of Veterinary Medicine, University College Dublin, Dublin, Ireland

**Keywords:** bacterial infection, feline cervicofacial neisseriosis, feline medicine, feline pulmonary neisseriosis, lymphadenopathy, hyperglobulinemia

## Abstract

An 11-year-old male neutered domestic shorthair cat underwent investigations for right mandibular and prescapular lymph node enlargement and weight loss. Cytology of the mandibular and prescapular lymph nodes revealed marked pyogranulomatous inflammation and mild neutrophilic, non-septic inflammation, respectively. Excisional biopsy of both lymph nodes was performed. Histopathology of the mandibular lymph node revealed severe pyogranulomatous inflammation, with prominent Splendore–Hoeppli phenomenon. Histopathology of the prescapular lymph node was consistent with reactive lymphoid hyperplasia. A *Neisseria* species was cultured from tissue samples obtained from both lymph nodes, with sensitivity testing confirming susceptibility to amoxicillin-clavulanate. After a 4-week course of amoxicillin-clavulanate (20 mg/kg PO q12h), clinical signs resolved. The cat was clinically well with no evidence of recurrence of infection 2.5 years after presentation.

## Case description

An 11-year-old male neutered domestic shorthair cat was presented to a university veterinary referral hospital for investigation of a right mandibular mass and weight loss. The cat was kept indoors only and had no relevant medical history, except for 2 dental extractions 3 years earlier due to periodontal disease. The mass (Figure S1) and weight loss were first noted 4 weeks before presentation to the referral hospital, at which time the primary care veterinarian drained approximately 10 mL of serosanguinous fluid from the mass and administered a subcutaneous injection of 1 mg/kg cefovecin (Convenia, Zoetis, Louvain-la-Neuve, Belgium) to treat a presumptive abscess. The mass subsequently recurred, prompting referral for further investigation.

On presentation, the cat was bright, alert, and responsive. A firm bilobed mass, measuring 5.1 cm × 3.6 cm × 3.4 cm was palpable in the region of the right submandibular lymph node. Moderate right prescapular lymph node enlargement was also noted. Mild generalized periodontal disease was present. Heart rate was 180 beats per minute, respiratory rate was 21 breaths per minute and rectal temperature was 101.9°F. Body condition score was 3/9, there was mild muscle loss, and the cat weighed 4.0 kg. No other abnormalities were detected on physical examination.

Complete blood count identified a mild mature neutrophilia of 16 000/μL (reference interval [RI], 2500-12 500/μL) and a mild monocytosis of 1070/μL (RI, 40-850/μL). Serum biochemistry identified marked hyperglobulinemia of 7.5 g/dL (RI, 2.4-4.0 g/dL) alongside a total protein of 10.2 g/dL (RI, 5.9-7.8 g/dL) and normal serum albumin of 2.7 g/dL (RI, 2.5-3.5 g/dL), with minor elevations in ALT and CK activity and blood glucose concentration. Serum potassium and phosphorus concentrations were lower than the laboratory RIs, at 3.57 mmol/L (RI, 4-4.5 mmol/L) and 1.30 mmol/L (RI, 1.40-2.5 mmol/L), respectively. The hyperglobulinemia was confirmed to be a polyclonal gammopathy on serum protein electrophoresis. Total T4 was normal at 2.46 μg/dL (RI, 1.17-3.88 μg/dL). Serum folate and cobalamin concentrations were 12.6 μg/L (RI, 8.2-13.5 μg/L) and 598 ng/L (RI, > 275 ng/L), respectively. Serum submitted for feline leukemia virus (FeLV) and feline immunodeficiency virus (FIV) serology (SNAP Combo FeLV Antigen/FIV Antibody Test [bidirectional flow ELISA], Idexx Laboratories) was negative for both agents.

Cytology of the right mandibular lymph node revealed marked pyogranulomatous inflammation. Ziehl–Neelsen (ZN) and PAS staining of the right mandibular lymph node aspirate slides were negative for the presence of acid-fast bacteria and fungal elements, respectively. Cytology of fine-needle aspirates of the right prescapular lymph node was consistent with mild neutrophilic, non-septic inflammation. Thoracic radiographs identified a small soft tissue opacity, measuring 2.3 mm × 2.1 mm, superimposed over the sixth rib, visible on both the DV and lateral projections. Abdominal ultrasonography identified mild splenomegaly but no other abnormalities. Cytology of hepatic and splenic fine needle aspirates was consistent with normal liver and spleen, respectively.

**Figure 1:**
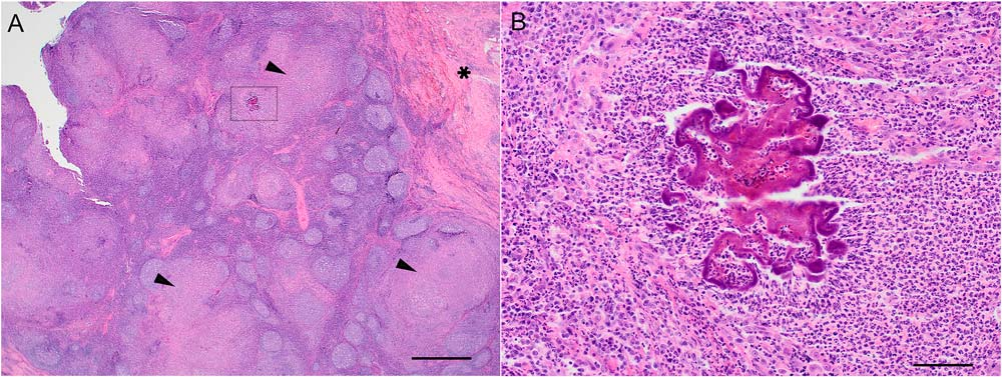
Photomicrograph of the submandibular lymph node of an 11-year-old cat with pyogranulomatous lymphadenitis caused by *Neisseria animaloris* infection. (A) Infiltrating the lymph node and the surrounding tissue are multifocal to coalescing pyogranulomas (arrowheads). There is fibrosis in the connective tissue (^*^) and multifocal perivascular plasma cell infiltrates. (B) The center of the pyogranulomas (magnification of box) consists of bright eosinophilic radiating material (Splendore–Hoeppli phenomenon) and neutrophils. Macrophages are present at the periphery. Hematoxylin and eosin stain. Scale bar: (A) 875 μm, (B) 50 μm.

Excisional biopsy of the right mandibular and right prescapular lymph nodes was subsequently performed under general anesthesia. Histopathology of the right mandibular lymph node showed severe pyogranulomatous inflammation, with prominent Splendore–Hoeppli phenomenon (Figure 1). Histopathology of the right prescapular lymph node was consistent with reactive lymphoid hyperplasia. Staining with ZN, PAS, and Gram stain was performed on both excised lymph nodes; no bacteria or fungal elements were identified in either node. Aerobic and anaerobic bacterial culture were performed on both excised nodes. A light growth of a slow growing *Neisseria* species was isolated from the right mandibular lymph node after direct plating on blood agar (Colombia blood agar base, Oxoid UK, supplemented with 5% sheep blood), incubated at 98.6°F. No growth was isolated from the right prescapular lymph node on the original culture, however a moderate growth of *Neisseria* species was also noted on tryptone soya broth (Oxoid UK) enrichment culture, incubated at 98.6°F, from this lymph node. The organism grew only under enriched CO_2_ atmospheric conditions and produced yellow colonies; short Gram-negative rods, resembling diplococci, were observed on Gram staining. The isolate was susceptible to marbofloxacin, enrofloxacin, gentamicin, trimethoprim-sulfamethoxazole, tetracycline, and amoxicillin-clavulanic acid on Kirby–Bauer disc diffusion testing. It was resistant to cephalothin. Definitive speciation was not possible using the VITEK 2 system (BioMérieux, Marcy-l’Étoile, France); however, identification was given as either *Neisseria animaloris* or *Neisseria zoodegmatis*.

Amoxicillin-clavulanate (Noroclav, Norbrook Laboratories, Monaghan, Ireland) was administered at a dose of 20 mg/kg PO q12h as the primary medical treatment for *Neisseria* lymphadenitis. The cat was discharged 2 days postoperatively, with instructions to continue administration of amoxicillin-clavulanate for a total of 4 weeks, and to administer meloxicam (Loxicom 0.5 mg/mL oral suspension for cats, Norbrook Laboratories, Monaghan, Ireland) at a dose of 0.05 mg/kg PO q12h for 4 days.

On recheck examination 4 weeks later, the owners reported that the cat had been clinically normal since the time of discharge, apart from moderate serosanguinous discharge from the right mandibular lymph node surgical site which had resolved within 1 week of discharge from hospital. Physical examination at this time revealed no new abnormalities. On hematology, the previous neutrophilia had fully resolved (neutrophil count 8100/μL; RI, 2500-12 500/μL), as had the previous hyperglobulinemia (3.2 g/dL; RI, 2.4-4.0 g/dL). The cat was clinically well 2.5 years after the time of initial presentation.

## Discussion

*Neisseria* spp. are Gram-negative bacteria belonging to the family *Neisseriaceae* that usually occur on the mucous membranes of humans and animals.^1–5^  *Neisseria gonorrhoeae*, which causes gonorrhea, and *N meningitidis*, which causes meningitis and sepsis, are prominent pathogenic *Neisseria* species in humans.^1^ In cats, *Neisseria* species—including N animaloris and N zoodegmatis (previously known as Centers for Disease Control and Prevention [CDC] group eugonic fermenter (EF)-4a and (EF)-4b, respectively),^6^ are usually considered to be commensals of the oral cavity of dogs and cats. However, they uncommonly cause disease in both animals and humans.^7–33^ Most zoonotic cases have been associated with local wound infections following cat or dog bites.^27–33^

The majority of cases of clinical disease associated with *Neisseria* spp. in cats in the literature can be classified into 1 of 2 groups: localized disease affecting the face, head, or neck^10^^,^^11^ (which the authors term feline cervicofacial neisseriosis [FCN]) and cases of severe pneumonia^12–24^ (which the authors term feline pulmonary neisseriosis [FPN]). In addition to the cat in this case report, 2 other cases of FCN are reported.^10^^,^^11^ As with this case, both other cases required bacterial culture of surgically obtained biopsies for diagnosis, and both cases successfully resolved with a combination of surgery and prolonged administration of antibiotics. Cases of FPN are more commonly reported than cases of FCN.^12–24^ Almost all described FPN cases presented with acute onset dyspnea due to severe pneumonia, and are characterized by rapid clinical deterioration, resulting in death or euthanasia.^12–21^ Four reported cases of successful medical treatment of FPN are described.^22–24^ Radiographically, the reported lung pattern findings of FPN are variable, but are most consistently described as discrete multifocal nodular densities, lung consolidation, or a combination of both of these findings, which have led authors to hypothesize a hematogenous origin of the pneumonia.^13^^,^^16^^,^^24^  *Neisseria* spp. was isolated in pure culture from the liver of a cat with chronic weight loss,^25^ a cat with pyothorax,^26^ a cat with pyopericardium,^27^ and have also been implicated in cases of keratitis, retrobulbar abscessation, otitis, sinusitis, and an infected fracture, although the clinical outcomes were not published in all of these cases.^18^

The cat in this case report developed FCN despite apparently being immunocompetent and having no known history of cervicofacial trauma, in line with other reported cases of this form of the disease.^10^^,^^11^ The authors hypothesize that FCN in this case was caused by translocation of the *Neisseria* species from the oral cavity to the right mandibular lymph node, with subsequent spread to the right prescapular lymph node. Although there was no known history of trauma that could have facilitated this translocation, it is possible that evidence of a prior internal or external penetrating injury might have healed by the time of examination, and thus might no longer have been evident. Alternatively, the concurrent periodontal disease in this cat could have been a factor allowing bacterial translocation; although the periodontal disease in this case was not severe, concurrent gingivitis has been reported in several cats with FPN.^20^ The cat in this case had no recent history of receiving immunosuppressive medications and tested negative for FIV and FeLV at presentation.

Moderate to marked unilateral mandibular lymph node enlargement is documented in several cats with FPN,^13^^,^^21^ and, in the one reported case of pyopericardium caused by *Neisseria* spp., an abscess in the cervical region was noted 3 weeks before the onset of signs of respiratory disease.^27^ These reports raise the possibility that local infection of the mandibular lymph nodes or cervical subcutaneous tissues might precede systemic involvement in some cases, and that FPN could be a possible sequela of unresolved FCN; local infection in the cervicofacial region might represent a source of repeated subclinical bacteremia, with subsequent hematogenous dissemination to the lungs.

Hyperglobulinemia is a commonly biochemical finding in cats with chronic infectious diseases including feline infectious peritonitis,^34^ FIV,^35^ leishmaniosis,^36^ atypical bacterial infections, such as bartonellosis,^37^ and chronic fungal infections.^38^^,^^39^ The hyperglobulinemia in such cases can generally be attributed to B-cell activation resulting from chronic antigenic stimulation.^35^^,^^39^ This case demonstrates that FCN should also be considered as a differential diagnosis for hyperglobulinemia in cats.

The histopathology in cats with *Neisseria* spp. infection commonly reveals marked necrosuppurative and histiocytic inflammation.^11^^,^^14^^,^^16^ This is often associated with intralesional and intrahistiocytic bacteria.^10^^,^^12^ In addition, as in the present case, the *Neisseria* species can elicit a Splendore–Hoeppli reaction and associated pyogranulomatous inflammation. This Splendore–Hoeppli reaction is characterized by aggregates of bacteria or other microorganisms, or biologically inert substances, surrounded by radiating star-like (asteroid) or club-shaped configurations of intensely eosinophilic material assumed to consist of antigen–antibody complexes, tissue debris, and fibrin.^40^ This reaction forms only when a unique dynamic balance between organism virulence and host response occurs and is described in one case of *Neisseria* pneumonia in a cat.^12^ Microorganisms that cause this phenomenon in cats include *Nocardia brasilensis*,^41^  *Microsporum canis*,^42^  *Streptococcus* spp.,^43^ and *Staphylococcus aureus*.^44^ Common locations affected are skin,^42^^,^^44^ lung,^12^ bone,^43^ and retroperitoneal space^41^ but to the authors knowledge this reaction has not been reported in a feline lymph node.

After the diagnosis of FCN in the cat in this case, it was decided to administer a 4-week course of an antimicrobial, in addition to surgery, due to the long duration of treatment required in other reported cases^10^^,^^11^ and due to concern for the possibility of development of severe pneumonia in the case of unresolved cervicofacial disease. After 4 weeks, it was decided to discontinue treatment as the cat was clinically normal and the previously documented hyperglobulinemia had resolved. The small lung opacity in this case was believed to most likely represent an incidental finding rather than a focus of pneumonia; however, unfortunately follow-up radiographs were not available. The cat remained clinically well 2.5 years after the time of initial presentation.

The *Neisseria* spp. that cause disease in cats can be slow-growing, and can be challenging to culture in laboratory conditions, sometimes requiring prolonged incubation or special media, or enrichment broth, to grow.^10^^,^^28^ Difficulty in identifying *N animaloris* and *N zoodegmatis* infection is described, with the bacteria often being misidentified as *Pasteurella* spp. or dismissed as a skin contaminant despite their pathogenic potential.^30^ Because of its small size and Gram reaction, the organism can be difficult to visualize on cytology or histopathology, necessitating culture to confirm infection.^11^ In addition, because of its relative biochemical unreactivity, molecular techniques are often required to confirm the identity of this bacterial species.^10^^,^^11^ Given that *Neisseria* spp. can be challenging to culture in commercial laboratory settings, techniques such as 16S rRNA sequencing might be useful to identify the presence of the bacteria in formalin-fixed tissue samples where the infection is suspected but bacterial culture is negative. It should, however, be noted that utilizing 16S rRNA sequencing to detect bacteria in formalin-fixed tissues is not without its own limitations—DNA damage during formalin-fixation and sample contamination can affect results.^45^ Due to the aforementioned challenges in identifying infection, it is possible that cases of disease related to *N animaloris*, *N zoodegmatis*, and related *Neisseria* species, are under-reported. This case demonstrates that FCN should be considered a differential diagnosis for pyogranulomatous lymphadenitis in cats, particularly if Splendore–Hoeppli phenomenon is present, even if bacteria are not visualized on histopathology. Histopathology and prolonged bacterial culture might be required to obtain a diagnosis. Prompt and prolonged antibiotic treatment is recommended, to resolve the infection and to prevent the possibility of progression to FPN.

## Supplementary Material

Supplementary_Figure_1_caption_aalag076

Suppl_Figure_1_aalag076

## Off-label antimicrobial declaration

The authors declare no off-label use of antimicrobials.

## Institutional animal care and use committee or other approval declaration

The authors declare no institutional animal care and use committee or other approval was needed.

## Abbreviations

CDC: Centers for Disease Control and Prevention
EF: eugonic fermenter
FCN: feline cervicofacial neisseriosis
FeLV: feline leukemia virus
FIV: feline immunodeficiency virus
FPN: feline pulmonary neisseriosis
RI: reference interval
ZN: Ziehl–Neelsen
