# Correction to: Pyogranulomatous lymphadenitis with Splendore–Hoeppli phenomenon caused by *Neisseria* species in a domestic shorthair cat

**DOI:** 10.1093/jvimsj/aalag180

**Published:** 2026-07-28

This is a correction to: Myles McKenna, Finola C Leonard, Hanne Jahns, Kevin Murtagh, Pyogranulomatous lymphadenitis with Splendore–Hoeppli phenomenon caused by *Neisseria* species in a domestic shorthair cat, *Journal of Veterinary Internal Medicine*, Volume 40, Issue 3, May–June 2026, aalag076, https://doi.org/10.1093/jvimsj/aalag076

In the originally published version of this manuscript, several units of measurement were incorrectly formatted due to an error that occurred during copyediting.

Specifically, “mg/kg” appeared as “mg kg^−1^”, “/μL” as “μL^−1^”, “d/L” as “dL^−1^”, “mmol/L” as “mmol L^−1^”, “μg/L” as “μg L^−1^”, “ng/L” as “ng L^−1^”, “mg/mL” as “mg mL^−1^”, and “g/dL” as “g dL^−1^”.

These errors have now been corrected.

