# Supplementary material for: Pyogranulomatous lymphadenitis with Splendore–Hoeppli phenomenon caused by Neisseria species in a domestic shorthair cat
Source: J Vet Intern Med. 2026 Jun 3;40(3):aalag076. doi: 10.1093/jvimsj/aalag076 (PMC13231861; doi:10.1093/jvimsj/aalag076)
Supplement: Supplementary_Figure_1_caption_aalag076 [file supplementary_figure_1_caption_aalag076.docx]

**Supplementary Figure 1**: The cat at the time of presentation. Note the mass in the right mandibular area (arrow heads).
