# Supplementary figures and images for: Pyogranulomatous lymphadenitis with Splendore–Hoeppli phenomenon caused by Neisseria species in a domestic shorthair cat
Source: J Vet Intern Med. 2026 Jun 3;40(3):aalag076. doi: 10.1093/jvimsj/aalag076 (PMC13231861; doi:10.1093/jvimsj/aalag076)

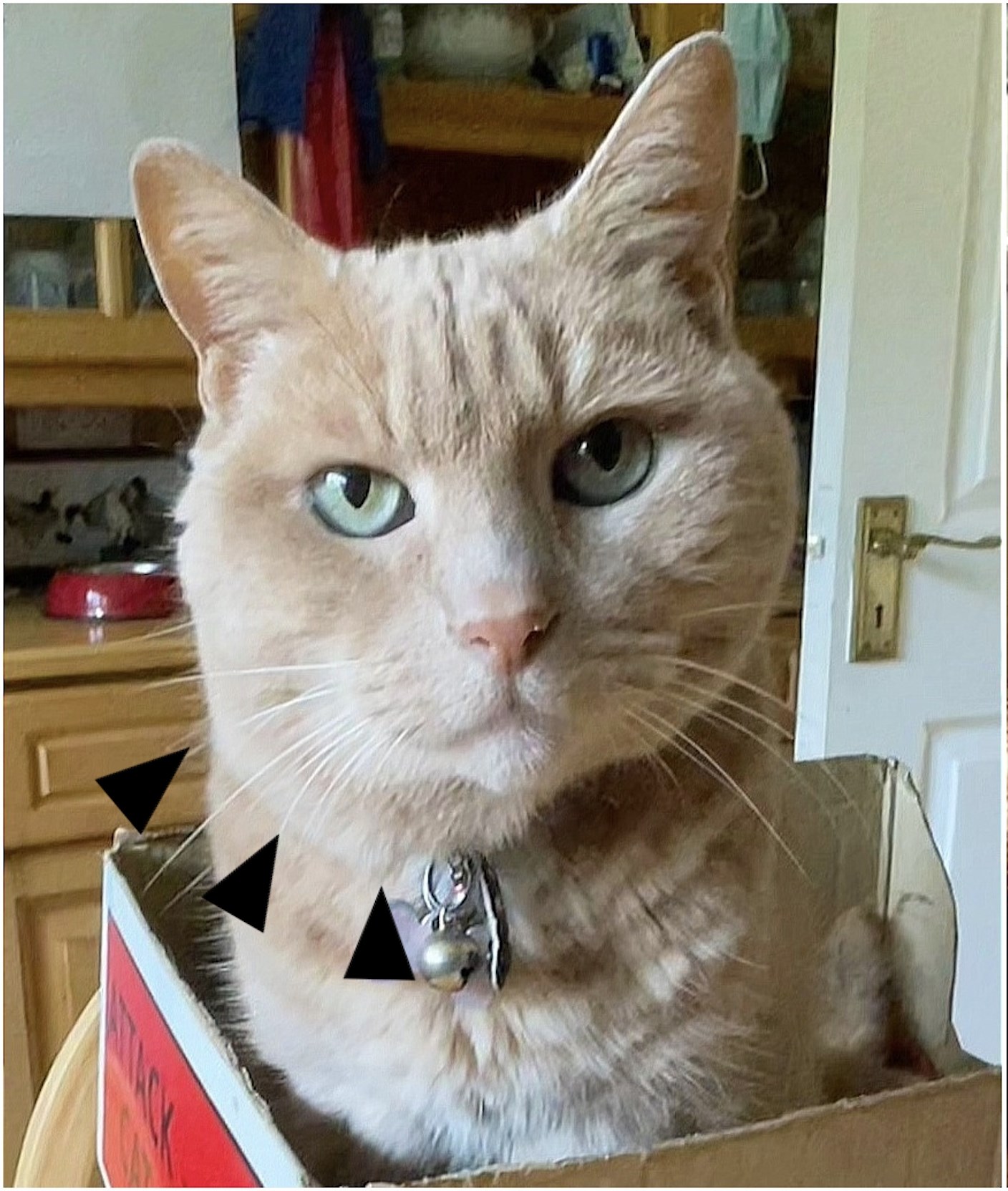

Supplement: Suppl_Figure_1_aalag076 [file suppl_figure_1_aalag076.jpeg]
